# Favourable Lifestyle Protects Cognitive Function in Older Adults With High Genetic Risk of Obesity: A Prospective Cohort Study

**DOI:** 10.3389/fnmol.2022.808209

**Published:** 2022-05-23

**Authors:** Huamin Liu, Zhenghe Wang, Lianwu Zou, Shanyuan Gu, Minyi Zhang, Daniel Nyarko Hukportie, Jiazhen Zheng, Rui Zhou, Zelin Yuan, Keyi Wu, Zhiwei Huang, Qi Zhong, Yining Huang, Xianbo Wu

**Affiliations:** ^1^Guangdong Provincial Key Laboratory of Tropical Disease Research, Department of Epidemiology, School of Public Health, Southern Medical University, Guangzhou, China; ^2^Baiyun Jingkang Hospital, Guangzhou, China

**Keywords:** cognitive function, obesity, genetic risk, lifestyle, older adults

## Abstract

The relationship between body mass index (BMI) and cognitive impairment remains controversial, especially in older people. This study aims to confirm the association of phenotypic and genetic obesity with cognitive impairment and the benefits of adhering to a healthy lifestyle. This prospective study included 10,798 participants (aged ≥ 50 years) with normal cognitive function from the Health and Retirement Study in the United States. Participants were divided into low (lowest quintile), intermediate (quintiles 2–4), and high (highest quintile) groups according to their polygenic risk score (PRS) for BMI. The risk of cognitive impairment was estimated using Cox proportional hazard models. Higher PRS for BMI was associated with an increased risk, whereas phenotypic obesity was related to a decreased risk of cognitive impairment. Never smoking, moderate drinking, and active physical activity were considered favourable and associated with a lower risk of cognitive impairment compared with current smoking, never drinking, and inactive, respectively. A favourable lifestyle was associated with a low risk of cognitive impairment, even in subjects with low BMI and high PRS for BMI. This study suggest that regardless of obesity status, including phenotypic and genetic, adhering to a favourable lifestyle is beneficial to cognitive function.

## Introduction

The association of body mass index (BMI) with cognitive impairment is still controversial. Results from meta-analysis have shown that high BMI in late-life markedly reduces the risk of cognitive impairment, and high BMI in mid-life increases the cognitive impairment risk (Qu et al., 2020). But a previous study also suggests that overweight or obese older individuals have an increased risk of cognitive impairment compared with those with normal weight (Wang et al., 2017). Phenotypic obesity is largely determined by heredity. Researchers have constructed a polygenic risk score (PRS) for BMI, a single score variable that summarises the effects of multiple single-nucleotide polymorphisms (SNPs) to evaluate the genetic risk of obesity (Locke et al., 2015). A study in Vietnam veteran men suggested that high PRS for BMI was associated with an increased risk of late-life cognitive impairment. However, this study used a small sample size and a special type of population (Xian et al., 2020). The PRS for BMI also increases the risk of depression, a disease with similar pathology to cognitive impairment (Avinun and Hariri, 2019). In addition, BMI and cognitive function share genetic contributions (Marioni et al., 2016). On the basis of these findings, we hypothesised that high PRS for BMI is positively related to cognitive impairment in the general older individuals.

Adhering to favourable lifestyles, such as no smoking, moderate drinking, and physical activity, has been shown to reduce the risk of several diseases in participants with high genetic risk as measured by PRS, including dementia, cardiovascular disease, diabetes, and cancer (Langenberg et al., 2014; Khera et al., 2016; Rutten-Jacobs et al., 2018; Lourida et al., 2019; Jin et al., 2020). It is worth noting that epigenetic mechanisms may also be involved in mediating the effects of lifestyle on phenotype, including cognitive impairment (Alegría-Torres et al., 2011). Therefore, the interaction between genetics and lifestyle helps us in identifying the combined risk of disease. However, the association between drinking and cognitive impairment remains controversial. Light to moderate drinking is associated with a lower risk of cognitive impairment and a consistently higher cognitive trajectory compared with never drinking in older United States adults (Zhang et al., 2020). The study from United Kingdom Biobank also found that any type of alcohol consumption in daily diet appeared to improve long-term cognitive performance (Klinedinst et al., 2020). However, in British middle-aged individuals, light drinking was not protective towards cognitive function, and even moderate drinking (14–21 units/week) results in higher odds of hippocampal atrophy and cognitive impairment compared with never drinking (Topiwala et al., 2017; Sabia et al., 2018).

In this study, we aimed to examine the relationship of PRS for BMI with incident cognitive impairment and further test the buffering of favourable lifestyle on cognitive risk in different genetic obesity groups, using a large population-based cohort from the Health and Retirement Study (HRS) conducted in the United States, especially when considering whether drinking is a favourable lifestyle. In addition, the association of BMI category with cognitive impairment was verified using our large sample given the conflicting results in previous studies.

## Materials and Methods

### Population

We used early release data from the HRS (public survey dataset, polygenic score dataset, and cognition dataset) sponsored by the National Institute on Aging (grant number: NIA U01AG009740) and conducted by the University of Michigan. The datasets can be downloaded from http://hrsonline.isr.umich.edu. The HRS is an ongoing longitudinal study with a nationwide representative older United States adults aged 50 years or above, together with their spouses of any age, and was biennially interviewed after its initiation in 1992 (wave 1). Although the HRS combined the AHEAD cohort, Children of Depression (CODA) cohort, War Baby (WB) cohort, Early Baby Boomer (EBB) cohort, and Mid Baby Boomer (MBB) cohort in wave 2, wave 4, wave 7, and wave 11, respectively, a small number of participants entered the cohort in other waves. Assessment methods for cognitive impairment have been modified since 1996 (wave 3). Therefore, the baseline of the participants who entered the survey in the first two waves was replaced by that in wave 3, for consistency of cognitive measurements in different waves (Zhang et al., 2020). We used data up to the 2014 wave, resulting in 9 waves of follow-up. A total of 12,090 European ancestry participants provided saliva samples for genotyping (Thompson et al., 2020). We excluded 514 individuals with incomplete baseline cognitive tests and 778 individuals with cognitive impairment at baseline. Finally, 10,798 subjects were eligible, and all these subjects completed at least two responses. The numbers of individuals at each stage of HRS were shown in a flow diagram (Supplementary Figure 1). The HRS was approved by the Institutional Review Board at the University of Michigan. All participants were asked to provide written informed consent.

### Polygenic Risk Score for Body Mass Index and Body Weight Status

For individuals who participated in the survey before 2006, saliva samples for half of the samples (randomly selected) were collected in 2006 (wave 8), and for the other half in 2008 (wave 9). For those who enrolled in the survey in 2010 (wave 10) and later, the saliva samples were collected randomly in 2010 (wave 10) and 2012 (wave 11), respectively. Genotyping was completed by HRS using the Illumina Omni-2.5 chip platform, conducted in the Center for Inherited Disease Research (Liebers et al., 2016). Quality control has been performed on genetic data, with exclusion criteria as follows: (1) minor allele frequency = 0 to remove mono-allelic markers; (2) missing call rate ≥ 2%; (3) discordant calls or Mendelian errors in duplicate subjects or family based samples; (4) Hardy-Weinberg equilibrium *P*-value < 10^–4^ in European or African samples; (5) sex difference in allelic frequency ≥ 0.2; (6) sex differences in heterozygosity > 0.3; (7) minor allele frequency < 0.01 (Laurie et al., 2010). Imputation of genetic data was performed according to the 1000 Genomes Project cosmopolitan reference panel phase 3 version 5.

Principal component analysis (PCA) has been performed by HRS to identify population group outliers and provide sample eigenvectors as covariates in the statistical model used for association testing to adjust for possible population stratification. These are referred to as “ancestry-specific PCs.” HRS has examined the predictive ability and the variability and co-variability in PRS and included all available SNPs in a PRS (i.e., not accounting for any linkage disequilibrium or *P*-value thresholding). All available SNPs in the PRS that overlap between the GWAS meta-analysis and the HRS genetic data were included (Ware et al., 2017). The BMI-related SNPs were based on the joint analysis of GWAS and MetaboChip meta-analysis conducted on 332,154 individuals across 2,554,623 SNPs, including 77 genome-wide significant loci; a second GWAS identified 20 more loci (97 genome-wide significant loci in total) (Locke et al., 2015). The PRS was calculated as the weighted sum of all SNPs, which were defined by the odds ratio or beta estimate from the GWAS

meta-analysis, and was standardised into mean = 0 and SD = 1. We used standardised PRS and categorised it into low (lowest quintile), intermediate (quintiles 2 to 4), and high (highest quintile) in this study.

Body weight status was determined by BMI, which was calculated on the basis of self-reported height and weight. According to the definitions of the World Health Organization, underweight, normal weight, overweight, class I obesity, class II obesity, or above were defined as BMIs < 18.5, 18.5–24.9, 25.0–29.9, 30–34.9, and ≥35 kg/m^2^, respectively. Underweight and normal-weight participants were classified into the same group due to the tiny minority of underweight participants.

### Assessment of Cognitive Impairment

Cognitive function was evaluated through the Telephone Interview for Cognitive Status, a modified and validated cognitive screening instrument (Xiang and An, 2015). A 27-point cognitive scale was used to assess cognitive function, which includes the following: recall 10 words from a list immediately and after 5 min (ranging from 0 to 20 points), subtract 7 from 100 serially five times (ranging from 0 to 5 points), and count backward as quickly as possible for 10 continuous numbers beginning with number 20. For backward count, answering correctly on the first and second attempts was, respectively, coded as 2 points and 1 point, and 0 point if both attempts failed. Respondents who scored 0–11 points were classified as cognitively impaired (Langa et al., 2017).

### Lifestyle

A favourable lifestyle was evaluated on the basis of smoking, drinking, and physical activity. Smoking status was divided into never, former, and current smoking. Drinking status was classified as never, former, current moderate, and excessive drinking according to established standards (Scott et al., 2020). Regular active physical activity was defined as at least two times a week for light, moderate, or vigorous physical activity. Two patterns of favourable lifestyle were defined in this study, in which current moderate drinking was initially defined as favourable, followed by never drinking. The details of the definitions are presented in Supplementary Table 1. The number of favourable lifestyles ranged from 0 to 3 based on the three lifestyles mentioned above.

### Covariates

Conventional factors, including baseline age, gender, education, marital status, and family wealth per capita, were collected by the structured questionnaires. Education was categorised as below high school, high school graduate, and college or above. Marital status was categorised as married, married but living alone, partnered, and never married. Family wealth per capita was classified into <$20,000, $20,000–50,000, and >$50,000. The HRS team imputed unreasonable or missing data using the multiple imputation method and provided a user-friendly subset. So the covariates were complete.

### Statistical Analysis

Baseline age was normally distributed and presented as a mean with SD and was compared by analysis of variance across three classifications of PRS for BMI. Other categorical variables were shown as frequencies with percentages. The chi-squared test was used for the analysis of unordered categorical variables, and the non-parametric Kruskal–Wallis test was used for ordered categorical variables.

The Cox proportional hazard models were used to test the association of PRS for BMI, body weight status, and lifestyle with incident cognitive impairment, represented by the hazard ratio (HR) and 95% confidence interval (CI). The follow-up time was from the year of baseline to the year of follow-up endpoint (occurrence of death, lost to follow-up, cognitive impairment, or observation until 2014, whichever came first). We constructed three models. The model 1 unadjusted any covariate; the model 2 adjusted for baseline age and sex; and the model 3 adjusted for baseline age, sex, lifestyle, education level, marital status, family wealth per capita, body weight status, and PRS for BMI. The top five PCs were also adjusted in the models of PRS and cognitive impairment according to the recommendations of the HRS genetic data guidelines. Sensitivity analyses were conducted through the three following methods: (1) grouping obese participants into class I and class II obesity; (2) excluding subjects who were underweight, and (3) categorising the PRS variable as quintiles in models. The models satisfied the assumption of proportional hazards after assessment using the Schoenfeld residuals technique.

The Cox proportional hazard model tests were repeated for lifestyle and cognitive impairment stratified by PRS for BMI or body weight status, respectively. In addition, the associations between the combination of lifestyle and PRS for BMI or body weight status and cognitive impairment were further evaluated. All statistical tests were two-sided. To maximise the likelihood of reporting true findings, we used Bonferroni correction to adjust for multiple testing of phenotypic BMI and PRS for BMI, in which the significance level was set at 0.05/the number of tests, and *P* values less than 0.05 were considered of suggestive significance. The significance level was still 0.05 for lifestyle and stratification analysis. Statistical analyses were performed using SAS software version 9.4 (SAS Institute, Inc., Cary, NC, United States).

## Results

### Characteristics of Participants

Amongst the 10,798 included participants [mean age (SD), 58.3 (8.7); 41.2% men], 3,297 (30.5%) cases were recorded over 122,232 person-years [median (interquartile range): 12 (6–16) years] of follow-up. Individuals with a high PRS for BMI were likely to be young and obese and had a low education level and less family wealth. The high PRS for BMI group had more smokers but fewer drinkers than those of the other groups. The distribution of sex, marital status, and physical activity status was similar amongst groups of PRS for BMI (Table 1).

**TABLE 1:** Characteristics of the participants stratified by PRS for BMI.

Characteristics	Total	PRS for BMI			*P*
		Low	Intermediate	High	
*n*	10798	2158	6482	2158	
Age (year ± SD)	58.3 ± 8.7	58.8 ± 8.9	58.3 ± 8.7	58.0 ± 8.3	0.004
Male, *n* (%)	4443 (41.2)	901 (41.8)	2646 (40.8)	896 (41.5)	0.692
Body weight status, *n* (%)					<0.001
Underweight	95 (0.85)	34 (1.6)	52 (0.8)	9 (0.4)	
Normal weight	3644 (34.3)	1046 (48.5)	2142 (33.1)	456 (21.1)	
Overweight	4351 (40.3)	807 (37.4)	2687 (41.5)	857 (39.7)	
Class I obesity	1830 (17.2)	209 (9.7)	1108 (17.1)	513 (23.8)	
Class II obesity or above	878 (8.2)	62 (2.9)	493 (7.6)	323 (15.0)	
Education, *n* (%)					<0.001
Below high school	1528 (14.2)	246 (11.4)	932 (14.4)	350 (16.2)	
High school graduate	3473 (32.2)	712 (33.0)	2044 (31.5)	717 (33.2)	
College or above	5797 (53.7)	1200 (55.6)	3506 (54.1)	1091 (50.6)	
Marital status, *n* (%)					0.426
Married	8166 (75.6)	1666 (77.2)	4886 (75.4)	1614 (74.8)	
Partnered	547 (5.1)	102 (4.7)	325 (5.0)	120 (5.6)	
Married but living alone	1777 (16.5)	335 (15.5)	1088 (16.8)	354 (16.4)	
Never married	308 (2.9)	55 (2.6)	183 (2.8)	70 (3.2)	
Family wealth per capita, *n* (%)					0.002
<20,000 $	4373 (40.5)	830 (38.5)	2593 (40.0)	950 (44.0)	
20,000– 50,000 $	4492 (41.6)	928 (43.0)	2691 (41.5)	873 (40.5)	
>50,000 $	1933 (17.9)	400 (18.5)	1198 (18.5)	335 (15.5)	
Smoking, *n* (%)					0.002
Never smoking	4712 (43.6)	1009 (46.8)	2819 (43.5)	884 (41.0)	
Former smoking	4045 (37.5)	771 (35.7)	2446 (37.7)	828 (38.4)	
Current smoking	2041 (18.9)	378 (17.5)	1217 (18.8)	446 (20.7)	
Drinking, *n* (%)					<0.001
Never drinking	3756 (34.8)	742 (34.4)	2260 (34.9)	754 (34.9)	
Former drinking	2306 (21.4)	393 (18.2)	1401 (21.6)	5123 (23.7)	
Moderate drinking	3254 (30.1)	701 (32.5)	1929 (29.8)	624 (28.9)	
Excessive drinking	1482 (13.7)	322 (14.9)	892 (13.8)	268 (12.4)	
Physical activity, *n* (%)					0.067
Inactive	1951 (18.1)	366 (17.0)	1176 (18.1)	409 (19.0)	
Two or more light	1892 (17.5)	352 (16.3)	1133 (17.5)	407 (18.9)	
Two or more moderate	3853 (35.7)	775 (35.9)	2330 (36.0)	748 (34.7)	
Two or more vigorous	3102 (28.7)	665 (30.8)	1843 (28.4)	594 (27.5)	

*PRS, polygenic risk score; BMI, body mass index; SD, standard deviation.*

### Cumulative Incidence of Cognitive Impairment

**FIGURE 1:**
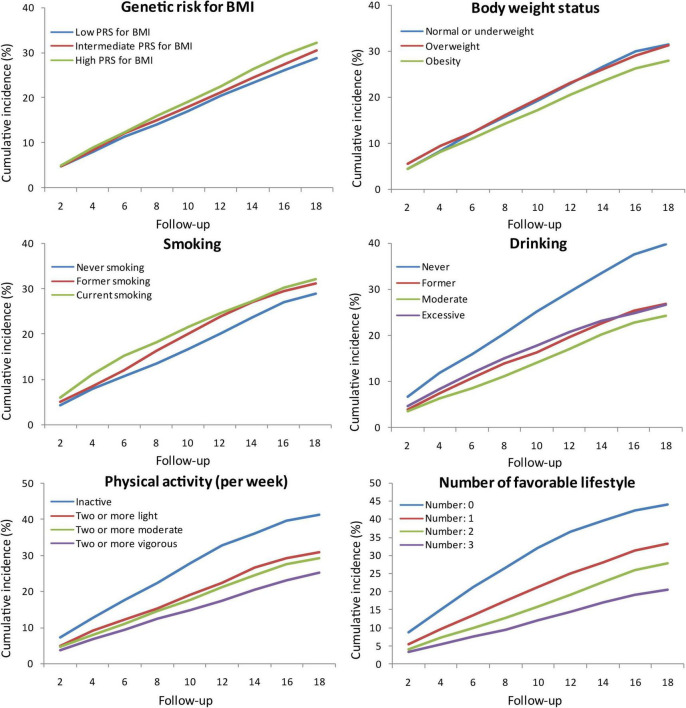
Cumulative incidence of cognitive impairment according to PRS for BMI, body weight status, and lifestyle. PRS, polygenic risk score; BMI, body mass index.

The cumulative incidence of cognitive impairment was lower in subjects with low PRS for BMI, obesity, never smoking, moderate drinking, and active physical activity. The cumulative incidence gradually decreased with the number of favourable lifestyles when moderate drinking was regarded as favourable (Figure 1). However, when never drinking was regarded as one of the favourable lifestyles, the cumulative incidence in participants with three favourable lifestyles outstripped that in those with one or two favourable lifestyles (Supplementary Figure 2).

### Polygenic Risk Score for Body Mass Index, Body Weight Status, and Incident Cognitive Impairment

**FIGURE 2:**
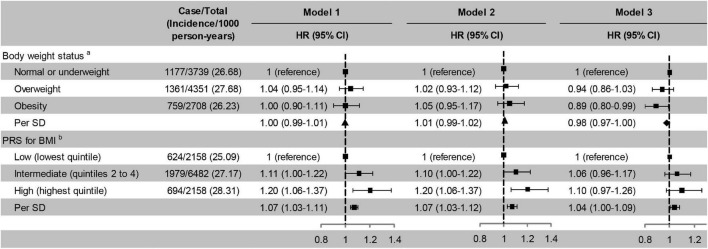
Risk of cognitive impairment according to body weight status and PRS for BMI. Model 1, unadjusted; Model 2, adjusted for baseline age and sex; Model 3, adjusted for smoking, drinking, physical activity, baseline age, sex, education, marital status, and family wealth per capita. ^a^PRS for BMI was also adjusted in model 3;^b^Five principal components and body weight status were also adjusted in model 3. ▲ Not significant; ◆ Significant. PRS, polygenic risk score; BMI, body mass index; HR, hazard ratio; CI, confidence interval; SD, standard deviation. The significance level was set at α = 0.025 (0.05/2).

Intermediate and high PRS for BMI were associated with a higher risk of cognitive impairment independent of five PCs, baseline age, and sex compared with low PRS for BMI. Further adjusted for education, marital status, and family wealth per capita, the association between high PRS for BMI and incident cognitive impairment was not statistically significant. But the risk of cognitive impairment increased with each additional SD of PRS in three models (Figure 2). The risk of cognitive impairment also increased with quintiles of PRS for BMI (Supplementary Table 2). Obesity was not significantly associated with a decreased risk of cognitive impairment compared with normal or underweight in model 1 and model 2, but significant in model 3, after adjusting for lifestyle and other covariates (Figure 2). In sensitivity analyses, the risk of cognitive impairment was increased with the weight grade (Supplementary Table 2).

### Lifestyle and Incident Cognitive Impairment

**FIGURE 3:**
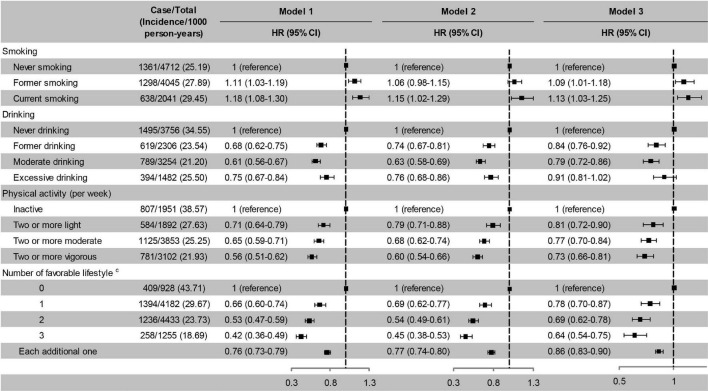
Risk of cognitive impairment according to lifestyle. Model 1, unadjusted; Model 2, adjusted for baseline age and sex; Model 3, adjusted for baseline age, sex, education, marital status, family wealth per capita, lifestyle, body weight status, and PRS for BMI. ^c^Moderate drinking was considered favourable. HR, hazard ratio; CI, confidence interval. The significance level was set at α = 0.05.

Former and current smoking were associated with an increased risk of cognitive impairment, while former and moderate drinking and active physical activity were associated with a decreased risk of cognitive impairment. The risk of cognitive impairment decreased monotonically with the number of favourable lifestyle when moderate drinking was considered favourable (Figure 3). When never drinking was considered to be a favourable lifestyle, the risk of cognitive impairment did not decrease with the number of favourable lifestyles (Supplementary Table 3).

### Lifestyle and Incident Cognitive Impairment Stratified by Polygenic Risk Score for Body Mass Index and Body Weight Status

**FIGURE 4:**
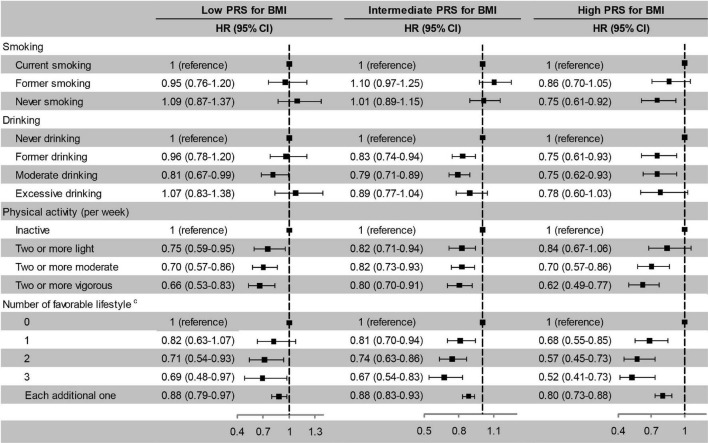
Risk of cognitive impairment according to lifestyle stratified by PRS for BMI. The model adjusted for smoking, drinking, physical activity, baseline age, sex, education, marriage, family wealth per capita, and body weight status. ^c^Moderate drinking was considered favourable. PRS, polygenic risk score; BMI, body mass index; HR, hazard ratio; CI, confidence interval. The significance level was set at α = 0.05.

**FIGURE 5:**
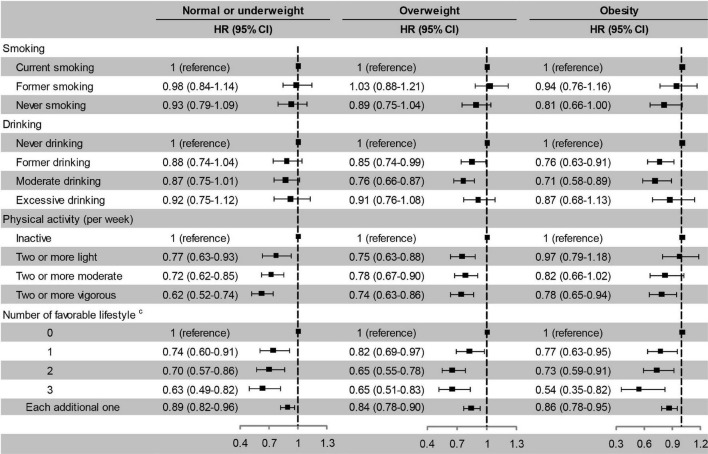
Risk of cognitive impairment according to lifestyle stratified by body weight status. The model adjusted for smoking, drinking, physical activity, baseline age, sex, education, marriage, family wealth per capita, and PRS for BMI. ^c^Moderate drinking was considered favourable. HR, hazard ratio; CI, confidence interval. The significance level was set at α = 0.05.

After stratified by PRS, the effects of favourable lifestyles on cognitive impairment were slightly stronger in the high PRS group (Figure 4). When stratified by weight status, the effects of smoking, drinking, and the number of favourable lifestyles on cognitive impairment were slightly stronger in the obesity group, and the effects of physical activity were slightly stronger in the normal or underweight group (Figure 5). However, when never drinking was considered to be one of the favourable lifestyles, the risk of cognitive impairment was not decreased with the number of favourable lifestyles in any stratification (Supplementary Tables 4, 5). There were no statistically significant interaction effects for lifestyle and PRS for BMI and body weight status.

### Cognitive Impairment and the Combination of Lifestyle and Polygenic Risk Score for Body Mass Index or Body Weight Status

**FIGURE 6:**
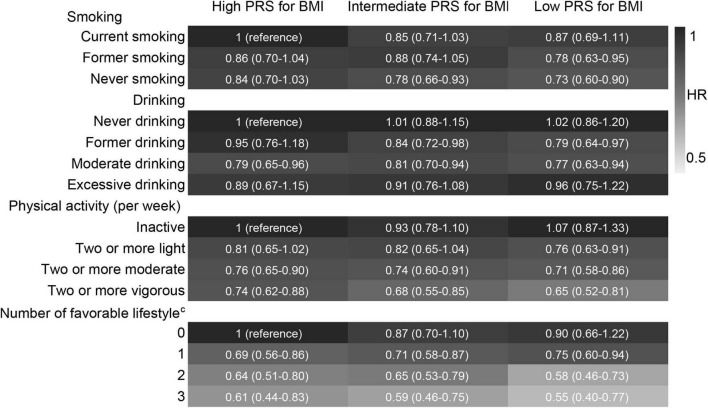
Risk of cognitive impairment according to the combination of lifestyle and PRS for BMI. HRs (95% CIs) were presented: light grey means protective. The model adjusted for smoking, drinking, physical activity, baseline age, sex, education, marriage, family wealth per capita, and body weight status. ^c^Moderate drinking was considered favourable. PRS, polygenic risk score; BMI, body mass index. The significance level was set at α = 0.05.

**FIGURE 7:**
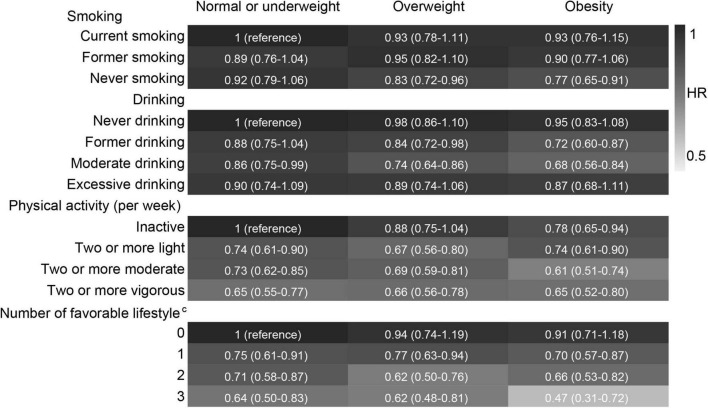
Risk of cognitive impairment according to the combination of lifestyle and body weight status. HRs (95% CIs) were presented: light grey means protective. The model adjusted for smoking, drinking, physical activity, age, sex, education, marriage, family wealth per capita, and PRS for BMI. ^c^Moderate drinking was considered favourable. The significance level was set at α = 0.05.

We have confirmed that high PRS, normal or underweight, current smoking, never drinking, and inactive physical activity were associated with a high risk of cognitive impairment according to the above results. Therefore, we combined high PRS/normal or underweight and each unfavourable lifestyle into a reference group in combination analyses. Compared with high PRS for BMI and unfavourable lifestyles, low PRS for BMI combined with never smoking, moderate drinking, and regular physical activity was associated with a lower risk of cognitive impairment. A higher number of favourable lifestyles combined with a lower PRS for BMI was associated with a lower risk of cognitive impairment (Figure 6). When never drinking was considered to be one of the favourable lifestyles, a lower extent of the decline in the risk of cognitive impairment was observed in subjects with three favourable lifestyles compared to those with one or two favourable lifestyles (Supplementary Figure 3). Compared with normal or underweight combined with unfavourable lifestyles, similar results were observed for lifestyles combined with overweight or obesity (Figure 7 and Supplementary Figure 4).

## Discussion

This large prospective study confirmed that high PRS for BMI was associated with an increased risk of cognitive impairment, whereas obesity was associated with a decreased risk of cognitive impairment. Favourable lifestyles, including never smoking, moderate drinking, and active physical activity, were associated with a low risk of cognitive impairment even in older adults with high PRS for BMI or low body weight status. A favourable lifestyle and low PRS or high body weight had combined effects on cognitive protection.

A favourable lifestyle can reduce the risk of cancer, cardiovascular disease, diabetes, and dementia despite high genetic risk (Cui et al., 2009; Langenberg et al., 2014; Khera et al., 2016; Rutten-Jacobs et al., 2018; Said et al., 2018; Lourida et al., 2019; Patten and Lein, 2019; Al Ajmi et al., 2020; Fan et al., 2020; Jin et al., 2020; Kehm et al., 2020; Ye et al., 2021). The findings in this study were partly consistent with those in previous studies. The difference was that the genetic risk of obesity, which is measured by the PRS for BMI, was used, but not the PRS for cognitive impairment. Cognitive impairment and obesity have shared genetic contributions, which imply that PRS for BMI is theoretically associated with cognitive impairment. But we also considered the obesity paradox of cognitive impairment, which contradicts genetic theory. Therefore, we used PRS for BMI but not PRS for cognitive impairment.

Generally, one risk factor involves multiple pathogeneses. Undoubtedly, genetic susceptibility to obesity implicates several pathways, including synaptic function, glutamate signalling, insulin secretion/action, energy metabolism, lipid biology, and adiposeness, which are all related to the central nervous system (Locke et al., 2015). Although the genetic risk of obesity provides new insight for the association between obesity and cognitive impairment, the influence of pleiotropic relations of gene may be present. Especially, the genetic interaction in multiple pathways forms a complex gene interaction network. On the one hand, PRS includes SNPs for various traits related to the obesity phenotype such as waist and hip circumference and cholesterol (Shabana et al., 2018). Those SNPs, related to a third variable such as cholesterol, may weaken the association between PRS for BMI and cognition. This is because we have found the protective role of cholesterol on cognition in our previous study (Liu et al., 2021). On the other hand, however, PRS for BMI based on 97 GWS loci has been demonstrated to be associated with cardiometabolic traits, schizophrenia, irritable bowel syndrome, and Alzheimer’s disease, and overlaps with genes and pathways implicated in neurodevelopment, all of which were associated with cognitive impairment (Locke et al., 2015). Therefore, PRS is limited to some extent due to pleiotropic relations.

The negative association of BMI with cognitive impairment in this study is consistent with another study using part of HRS data, in which the obese subjects had a better cognitive function (Bryant et al., 2014). But we found a positive association of PRS for BMI with cognitive impairment. This seems like an exciting result, which reminds us of the different potential risks of outcomes for participants with genetic and phenotypic obesity. Several possible reasons can explain the negative association of BMI with cognitive impairment. A higher BMI is associated with a higher level of insulin-like growth factor I (IGF-1), leptin hormone, and oestrogen, all of which are beneficial to cognition (Qu et al., 2020). In addition, the muscle loss is also combined with the decrease in BMI (Zhou et al., 2020), and the muscle loss is an indication of frailty, which increases the risk of cognitive impairment (Nishiguchi et al., 2015). The positive association of PRS for BMI with cognitive impairment is also found in Vietnam veteran men and Scotsmen (Marioni et al., 2016; Xian et al., 2020). Furthermore, Marioni et al. demonstrated that there are partial genetic overlaps between BMI and cognitive function (Marioni et al., 2016). However, the exact mechanism between obesity and cognitive impairment remains unclear, which leads to our inability to explain the opposite relationship of BMI and PRS with cognitive impairment.

Modifying lifestyle is easier than controlling disease, blood indicators, and genetic susceptibility, and so on. Never smoking and active physical activity are well-established favourable lifestyles for multiple health conditions. We also confirmed this in this study. However, there are still inconsistent opinions regarding drinking. For British individuals, drinking is considered harmful to cognition (Topiwala et al., 2017; Sabia et al., 2018). However, a dose-response meta-analysis of prospective studies found that light to moderate drinking is associated with a reduced risk of dementia (Xu et al., 2017). Our results indicate that moderate drinking is favourable for cognition, which is consistent with a study that investigated drinking and functional ability using HRS data (Scott et al., 2020). The risk of cognitive impairment did not decrease with the increased number of favourable lifestyles when never drinking was regarded as favourable, which further supports our opinion. Perhaps, the different definitions of drinking frequency or drinking amount contributed to these inconsistent results. In addition, abstention requires careful consideration. Abstention retains a residual effect of alcohol compared to never drinking. Abstention may also be chosen because of other health issues, and these issues can promote cognitive impairment. However, we could not identify the motivation of abstention using our data, so that we could not determine which self-abstainers have the same alcohol effect as current drinkers rather than due to other health issues. Thus, we modelled abstainers as a single group and defined current moderate drinking as a favourable lifestyle.

We grouped participants based on PRS and body weight status to verify the protection of a favourable lifestyle. The gradually decreased risk of cognitive impairment with the number of favourable lifestyles is similar to that of research on dementia using the United Kingdom Biobank data (Lourida et al., 2019). Previous similar studies did not show the risk of disease for each lifestyle combined with genetic risk, just the comprehensive score of multiple lifestyles. In this study, we showed both. The risk of cognitive impairment decreased more in high PRS stratification for each favourable lifestyle, especially smoking. That is, a favourable lifestyle brings greater benefit in a high-risk population. But for body weight status stratification, the results became irregular because we found the lowest risk of cognitive impairment for never smoking and moderate drinking in the obesity group (regarded as the low-risk group). After stratified analysis, we used combination variables to verify the combined effects of lifestyle and PRS/body weight status on cognition. The lowest risk of cognitive impairment was shown in favourable lifestyles (never smoking, moderate drinking, and vigorous physical activity) combined with low genetic risk or high BMI groups. This approach was widely used in previous studies, in which the findings were similar to ours (Khera et al., 2016; Rutten-Jacobs et al., 2018; Lourida et al., 2019; Jin et al., 2020).

Another noteworthy mechanism between lifestyle and cognitive impairment is the epigenetic mechanism. Different from SNP, the epigenome, such as DNA modification, histone modification, chromatin remodelling, and non-coding RNA, is dynamic, does not change the DNA sequence, but can change genome expression under exogenous influence (Alegría-Torres et al., 2011). It provides a suitable condition by which lifestyle affects cognitive function. Although our data cannot support an epigenetic mechanism for cognitive impairment, we believe that there must be epigenetic changes between lifestyle and cognitive impairment. This may also be a potential reason for the opposite association of PRS and BMI on cognitive impairment. Therefore, we encourage researchers to explore the interaction between epigenetic mechanisms and the environment on cognitive function in future studies.

This study has several limitations. First, the causal relationship between PRS for BMI and cognitive impairment cannot be determined based on the current observational study. Second, the biochemical factors were excluded from the covariates because the blood sample used for the biochemical factor test was collected between waves 8 and 11, which resulted in a large proportion of participants lacking the baseline biochemical indicator. Third, the cognition assessment did not involve the full range of cognitive functioning despite the reliability and validity of this assessment. The PRS for BMI and body weight status may have different effects on other specific dimensions of cognitive function. Fourth, a small number of underweight individuals were observed. The association of underweight with cognitive impairment remains unknown in the present population. Fifth, the GWAS of the PRS for BMI was based on midlife individuals, but BMI is dynamic. PRS may better reflect BMI in middle age. Finally, physical activity was only assessed through frequency, while the time of each type on a given day was not obtained, which resulted in assessment bias for the actual physical activity amount.

## Conclusion

Genetic obesity and phenotypic obesity were associated with cognitive impairment in opposite directions amongst European-ancestry older individuals. This study suggested that never smoking, moderate drinking, and active exercise were still associated with a low cognitive impairment risk even in older adults with high PRS for BMI.

## Data Availability Statement

Publicly available datasets were analysed in this study. This data can be found here: http://hrsonline.isr.umich.edu, University of Michigan.

## Ethics Statement

The HRS was approved by the Institutional Review Board at the University of Michigan. The participants provided their written informed consent to participate in this study. Ethical review and approval was not required for the study on human participants in accordance with the local legislation and institutional requirements. The patients/participants provided their written informed consent to participate in this study.

## Author Contributions

HL wrote the manuscript. KW, ZH, ZY, and JZ performed the data analysis. ZW, XW, and RZ drafted and critically revised the manuscript. LZ and SG provided clinical guidance. DH reviewed language. MZ made substantial interpretation. YH and QZ cleaned the data. XW contributed to the study concept and design and reviewed the manuscript. All authors contributed to the article and approved the submitted version.

## Conflict of Interest

The authors declare that the research was conducted in the absence of any commercial or financial relationships that could be construed as a potential conflict of interest.

## Publisher’s Note

All claims expressed in this article are solely those of the authors and do not necessarily represent those of their affiliated organizations, or those of the publisher, the editors and the reviewers. Any product that may be evaluated in this article, or claim that may be made by its manufacturer, is not guaranteed or endorsed by the publisher.
