# Supplementary material for: Favourable Lifestyle Protects Cognitive Function in Older Adults With High Genetic Risk of Obesity: A Prospective Cohort Study
Source: Front Mol Neurosci. 2022 May 23;15:808209. doi: 10.3389/fnmol.2022.808209 (PMC9169719; doi:10.3389/fnmol.2022.808209)
Supplement: Supplementary file 1 [file Table_1.DOCX]

**Favorable lifestyle protects** **cognitive function in older adults with** **high genetic risk of obesity:** **A** **prospective cohort study**

**Supplementary Materials:**


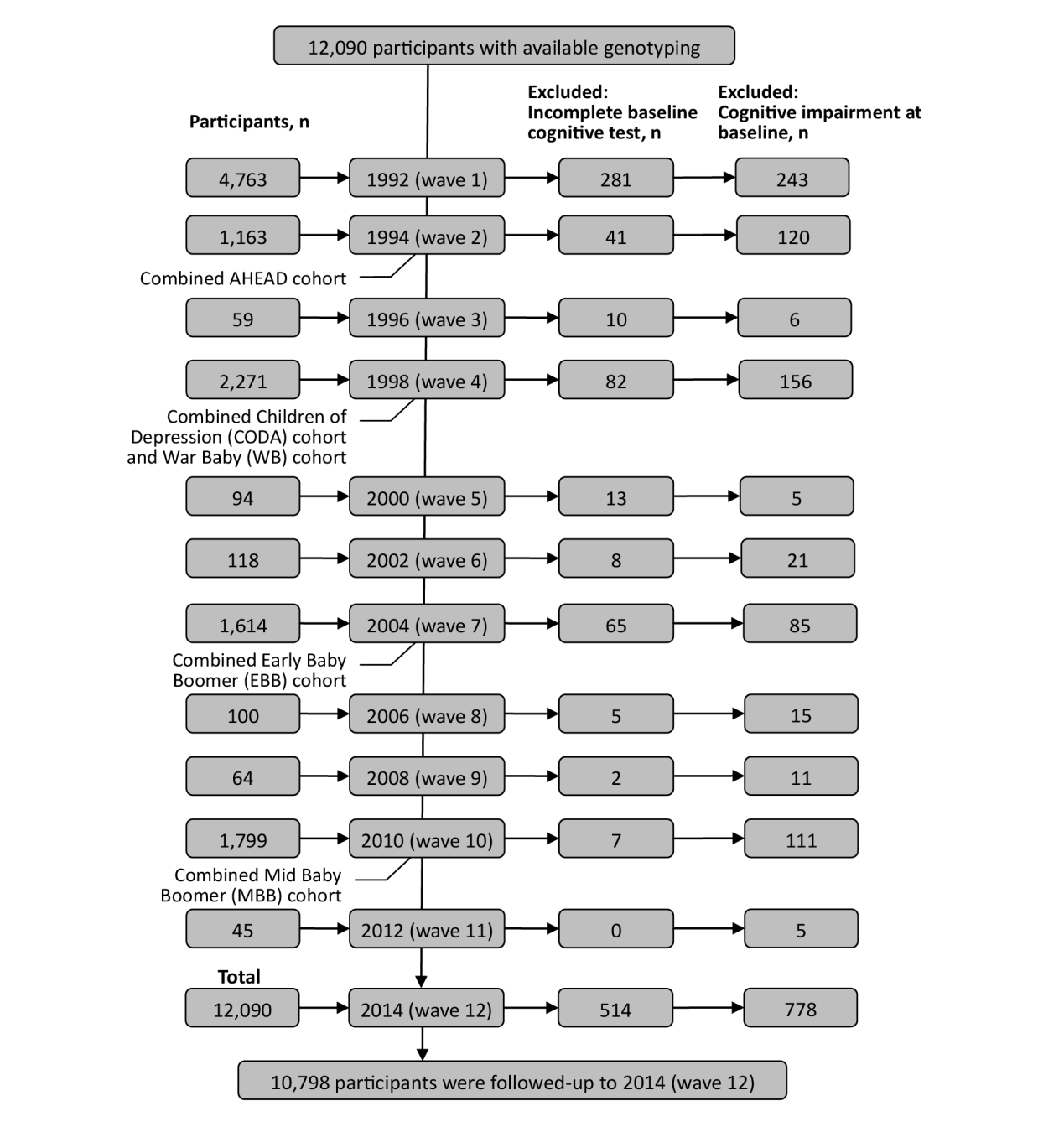


**Supplementary Figure S1** Flow diagram for the selection of the analyzed study sample

**Supplementary Table S1** Definitions of lifestyle and favorable lifestyle in the present study

| Factors | Definitions |
| --- | --- |
| **Smoking** |  |
| Never smoking | No smoking from birth to survey date |
| Former smoking | Had smoked from birth to the survey date, which was more than one year since last smoking |
| Current smoking | Started smoking at some time before the survey date and has continued until now or less than one year since last smoking |
| **Drinking** |  |
| Never drinking | No drinking from birth to survey date |
| Former drinking | Drinking from birth to the survey date, but no drinking within one year before the survey date |
| Moderate drinking | Current regular drinking, fewer than three drinks on a given day, and fewer than seven drinks in a week |
| Excessive drinking | Current regular drinking, and exceed moderate drinking |
| **Physical activity** |  |
| Inactive | Almost no physical activity of any type or less twice a week for any type of physical activity |
| Light physical activity | Including sports or activities that are mildly energetic, such as vacuuming, laundry and home repairs |
| Moderate physical activity | Including moderately energetic, such as gardening, cleaning the car, walking at a moderate pace, dancing, floor or stretching exercises |
| Vigorous physical activity | Including running or jogging, swimming, cycling, aerobics or gym workout, tennis, or digging with a spade or shovel |
| **Favorable lifestyle (pattern 1)** |  |
| Favorable smoking status | Never smoking |
| Favorable drinking status | Moderate drinking |
| Favorable physical activity | Light to vigorous physical activity at least twice a week |
| **Favorable lifestyle (pattern 2)** |  |
| Favorable smoking status | Never smoking |
| Favorable drinking status | Never drinking |
| Favorable physical activity | Light to vigorous physical activity at least twice a week |


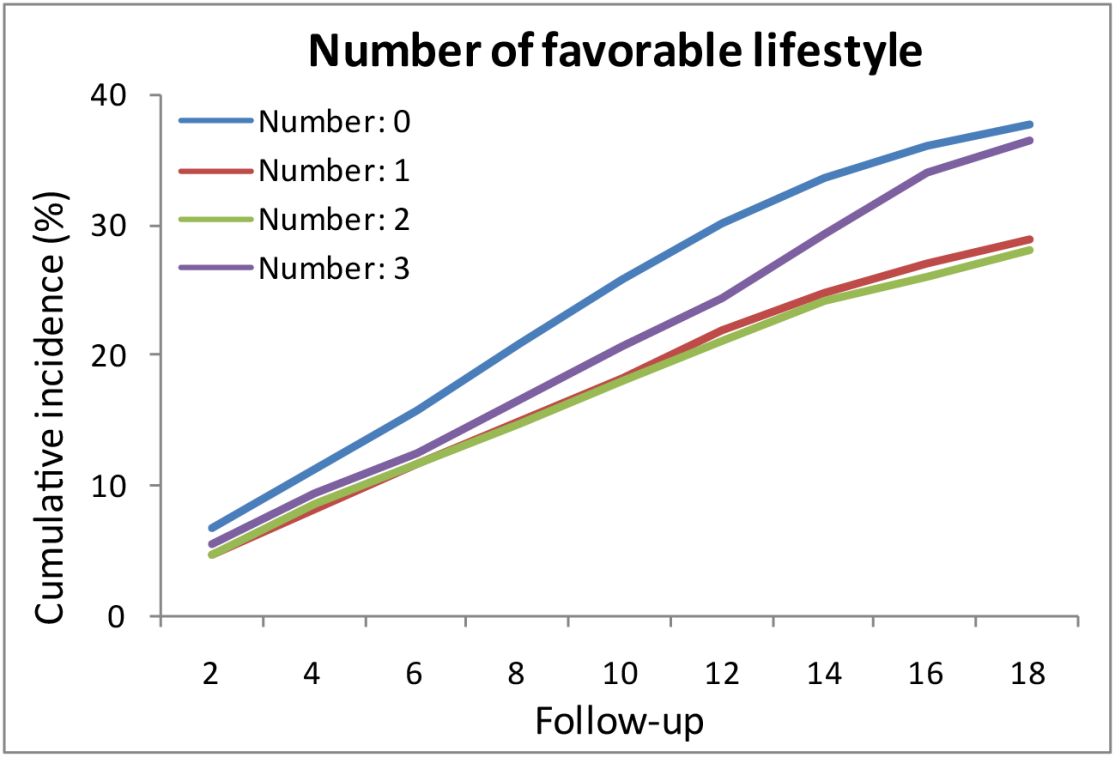


**Supplementary Figure S2** Cumulative incidence of cognitive impairment according to the number of favorable lifestyles (Never drinking was considered favourable)

**Supplementary Table S2** Sensitivity analysis of body weight status and PRS for BMI with cognitive impairment

|  | Model 1 | |  | Model 2 | |  | Model 3 | |
| --- | --- | --- | --- | --- | --- | --- | --- | --- |
|  | HR (95% CI) | *P* |  | HR (95% CI) | *P* |  | HR (95% CI) | *P* |
| Body weight status ^d^ |  |  |  |  |  |  |  |  |
| Normal or underweight | 1 (reference) |  |  | 1 (reference) |  |  | 1 (reference) |  |
| Overweight | 1.04 (0.97-1.13) | 0.292 |  | 1.02 (0.94-1.11) | 0.593 |  | 0.94 (0.87-1.02) | 0.139 |
| Class I obesity | 1.02 (0.92-1.12) | 0.777 |  | 1.04 (0.94-1.15) | 0.451 |  | 0.90 (0.81-0.99) | 0.045 |
| Class II obesity or above | 0.95 (0.82-1.10) | 0.495 |  | 1.08 (0.94-1.26) | 0.259 |  | 0.87 (0.75-0.98) | 0.038 |
| Body weight status ^d, e^ |  |  |  |  |  |  |  |  |
| Normal weight | 1 (reference) |  |  | 1 (reference) |  |  | 1 (reference) |  |
| Overweight | 1.05 (0.97-1.14) | 0.231 |  | 1.03 (0.95-1.11) | 0.491 |  | 0.95 (0.87-1.03) | 0.174 |
| Class I obesity | 1.02 (0.92-1.13) | 0.691 |  | 1.05 (0.94-1.16) | 0.383 |  | 0.90 (0.81-1.00) | 0.056 |
| Class II obesity or above | 0.96 (0.83-1.11) | 0.549 |  | 1.10 (0.95-1.27) | 0.226 |  | 0.87 (0.75-0.99) | 0.048 |
| PRS for BMI ^f^ |  |  |  |  |  |  |  |  |
| Quintile 1 | 1 (reference) |  |  | 1 (reference) |  |  | 1 (reference) |  |
| Quintile 2 | 1.07 (0.96-1.19) | 0.209 |  | 1.07 (0.96-1.19) | 0.225 |  | 1.08 (0.97-1.20) | 0.174 |
| Quintile 3 | 1.10 (0.99-1.23) | 0.075 |  | 1.10 (0.99-1.23) | 0.078 |  | 1.08 (0.97-1.20) | 0.177 |
| Quintile 4 | 1.14 (1.02-1.27) | 0.017 |  | 1.14 (1.02-1.27) | 0.018 |  | 1.12 (1.00-1.25) | 0.048 |
| Quintile 5 | 1.21 (1.08-1.35) | 0.001 |  | 1.20 (1.08-1.35) | 0.001 |  | 1.14 (1.02-1.28) | 0.023 |
| *P* for trend | 1.05 (1.02-1.07) | <0.001 |  | 1.05 (1.02-1.07) | <0.001 |  | 1.03 (1.01-1.06) | 0.021 |

Model 1, unadjusted;

Model 2, adjusted for age and sex;

Model 3, adjusted for age, sex, smoke, drink, physical activity, education, marriage, family wealth per capita.

^d^ PRS for BMI was also adjusted in model 3, the significance level was set α = 0.017 (0.05/3). ^e^ Underweight participants were excluded. ^f^ Five principal components and body weight status were also adjusted, the significance level was set α = 0.013 (0.05/4). HR, hazard ratio; CI, confidence interval.

**Supplementary Table S3** Association between the number of favorable lifestyle and cognitive impairment (Never drinking was considered favorable)

|  | Model 1 | |  | Model 2 | |  | Model 3 | |
| --- | --- | --- | --- | --- | --- | --- | --- | --- |
|  | HR (95% CI) | *P* |  | HR (95% CI) | *P* |  | HR (95% CI) | *P* |
| Number of favorable lifestyle |  |  |  |  |  |  |  |  |
| 0 | 1 (reference) |  |  | 1 (reference) |  |  | 1 (reference) |  |
| 1 | 0.70 (0.62-0.80) | <0.001 |  | 0.73 (0.64-0.83) | <0.001 |  | 0.78 (0.69-0.89) | <0.001 |
| 2 | 0.71 (0.63-0.81) | <0.001 |  | 0.75 (0.66-0.85) | <0.001 |  | 0.80 (0.70-0.91) | <0.001 |
| 3 | 0.82 (0.71-0.95) | 0.006 |  | 0.82 (0.71-0.95) | 0.008 |  | 0.85 (0.74-0.99) | 0.031 |
| Each additional one | 0.99 (0.95-1.03) | 0.623 |  | 0.99 (0.95-1.03) | 0.562 |  | 0.99 (0.95-1.03) | 0.536 |

HR, hazard ratio; CI, confidence interval. The significance level was set α = 0.05.

Model 1, unadjusted;

Model 2, adjusted for age and sex;

Model 3, adjusted for age, sex, education, marital status, family wealth per capita, body weight status and PRS for BMI.

**Supplementary Table S4** Association between the number of favorable lifestyle and cognitive impairment stratified by PRS for BMI (Never drinking was considered favorable)

|  | Low PRS for BMI | |  | Intermediate PRS for BMI | |  | High PRS for BMI | |
| --- | --- | --- | --- | --- | --- | --- | --- | --- |
|  | HR (95% CI) | *P* |  | HR (95% CI) | *P* |  | HR (95% CI) | *P* |
| Number of favorable lifestyle |  |  |  |  |  |  |  |  |
| 0 | 1 (reference) |  |  | 1 (reference) |  |  | 1 (reference) |  |
| 1 | 0.71 (0.54-0.94) | 0.015 |  | 0.86 (0.72-1.02) | 0.079 |  | 0.68 (0.52-0.88) | 0.004 |
| 2 | 0.73 (0.55-0.96) | 0.025 |  | 0.88 (0.74-1.05) | 0.164 |  | 0.67 (0.51-0.88) | 0.003 |
| 3 | 0.82 (0.61-1.12) | 0.208 |  | 0.91 (0.75-1.11) | 0.912 |  | 0.75 (0.55-1.02) | 0.069 |
| Each additional one | 0.99 (0.90-1.08) | 0.781 |  | 1.00 (0.95-1.06) | 0.976 |  | 0.95 (0.86-1.04) | 0.259 |

PRS, polygenic risk score; BMI, body mass index; HR, hazard ratio; CI, confidence interval. The model adjusted for age, sex, education, marital status, family wealth per capita and body weight status. The significance level was set α = 0.05.

**Supplementary Table S5** Association between the number of favorable lifestyle and cognitive impairment stratified by body weight status (Never drinking was considered favorable)

|  | Normal or underweight | |  | Overweight | |  | Obesity | |
| --- | --- | --- | --- | --- | --- | --- | --- | --- |
|  | HR (95% CI) | *P* |  | HR (95% CI) | *P* |  | HR (95% CI) | *P* |
| Number of favorable lifestyle |  |  |  |  |  |  |  |  |
| 0 | 1 (reference) |  |  | 1 (reference) |  |  | 1 (reference) |  |
| 1 | 0.67 (0.54-0.83) | <0.001 |  | 0.83 (0.68-1.01) | 0.064 |  | 0.88 (0.67-1.14) | 0.327 |
| 2 | 0.72 (0.58-0.89) | 0.003 |  | 0.83 (0.67-1.02) | 0.069 |  | 0.87 (0.67-1.14) | 0.315 |
| 3 | 0.74 (0.58-0.94) | 0.015 |  | 0.86 (0.68-1.08) | 0.197 |  | 1.00 (0.74-1.34) | 0.999 |
| Each additional one | 0.98 (0.91-1.05) | 0.542 |  | 0.98 (0.91-1.04) | 0.484 |  | 1.02 (0.93-1.11) | 0.723 |

PRS, polygenic risk score; BMI, body mass index; HR, hazard ratio; CI, confidence interval. The model adjusted for age, sex, education, marriage, family wealth per capita, and PRS for BMI. The significance level was set α = 0.05.


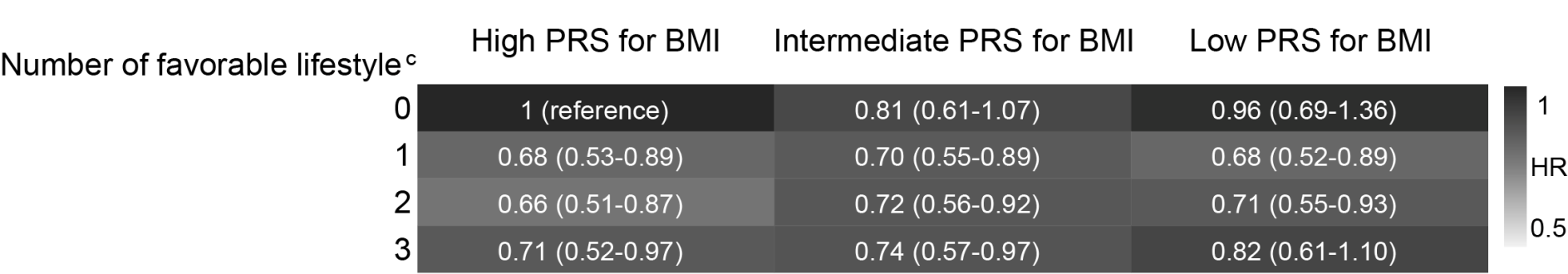


**Supplementary Figure S3** Risk of cognitive impairment according to the combination of the number of favorable lifestyles (Never drinking was considered favorable). HRs (95% CIs) were presented: light gray means protective. The model adjusted for age, sex, education, marriage, family wealth per capita, and body weight status. PRS, polygenic risk score; BMI, body mass index. The significance level was set α = 0.05.


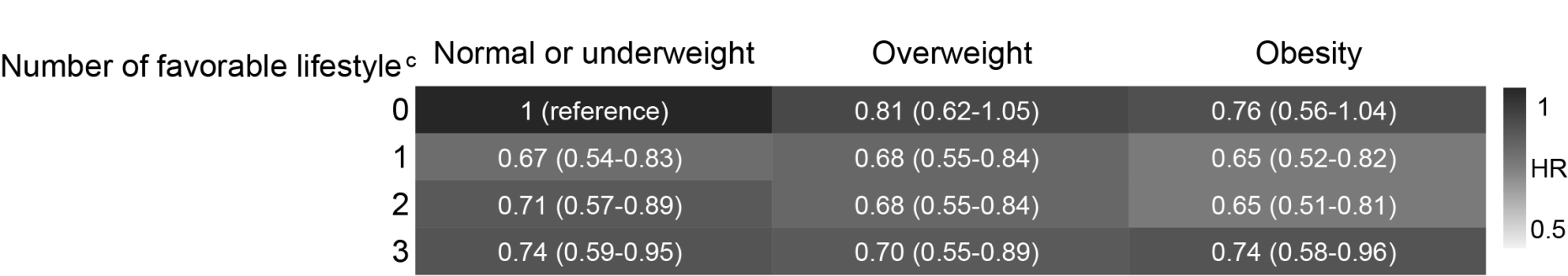


**Supplementary Figure S4** Risk of cognitive impairment according to the combination of the number of favorable lifestyles (Never drinking was considered favorable) and body weight status. HRs (95% CIs) were presented: light gray means protective. The model adjusted for age, sex, education, marriage, family wealth per capita, and PRS for BMI. The significance level was set α = 0.05.
